# 

**Published:** 1867-08

**Authors:** 


### CONTENTS.

ORIGINAL COMMUNICATIONS.

The Relationship of Matter .......... 327
Address of Wm. O. Kulp, D.D.S .......... 337
Extracting Teeth .......... 341

PROCEEDINGS OF SOCIETIES.

Michigan Dental Association .......... 344
Dental Association of Ontario .......... 352
Northern Ohio Dental Association .......... 356
Iowa State Dental Society .......... 357
Notice to Dentists .......... 362

EDITORIAL.

Hull's Benzine Gas Burner .......... 364
"One More Unfortunate." .......... 365
Cheap Science .......... 366
Therapeutic. .......... 366
How do You Get Along with Nitrous Oxyd .......... 367
The Rubber Question .......... 368
Missouri Dental College .......... 368
American Dental Association. .......... 369
Dental Chair .......... 369
A New Dental College .......... 370
Watt's Chemical Essays .......... 370
Societies .......... 370

